# Two Cases of External Auditory Canal Osteonecrosis in Patients on Antiresorptive Therapy for Osteoporosis

**DOI:** 10.1210/jcemcr/luad021

**Published:** 2023-02-27

**Authors:** Shejil Kumar, Terrence Diamond, Joanna Walton

**Affiliations:** Endocrinology Department, St George Public Hospital, Sydney, NSW 2217, Australia; St George Hospital Clinical School, University of New South Wales, Sydney, NSW 2217, Australia; Endocrinology Department, St George Public Hospital, Sydney, NSW 2217, Australia; Ear Nose & Throat Surgeon, ENT Sydney, Sydney, NSW 2027, Australia

**Keywords:** external auditory canal, osteonecrosis, denosumab, bisphosphonates, osteoporosis

## Abstract

Bisphosphonates and denosumab have demonstrated overwhelmingly favorable skeletal benefit/risk profile in managing postmenopausal osteoporosis. External auditory canal osteonecrosis is a rare skeletal complication of antiresorptives previously described in 11 patients with bisphosphonate exposure and 1 bisphosphonate-naïve patient on denosumab. We present 2 patients who developed external auditory canal osteonecrosis while taking antiresorptives for postmenopausal osteoporosis; a 79-year-old asymptomatic bisphosphonate-naïve woman with 2-year exposure to denosumab, and a 64-year-old woman with otalgia after 5 years of risedronate and 5 years of denosumab treatment. Neither patient had previous exposure to glucocorticoids or local radiotherapy. Otoscopy performed by an ear/nose/throat (ENT) surgeon revealed exposed areas of bone in external auditory canal in both patients. Computed tomography of temporal bones found no evidence of bone erosion. Bone turnover markers were suppressed. Both patients ceased denosumab and were managed conservatively, with stable external auditory canal findings after 12 months. Although external auditory canal osteonecrosis is a rare skeletal complication of antiresorptive use, development of localizing symptoms in the ear should alert physicians to this rare clinical entity and prompt ENT surgical referral for early diagnosis and initiation of management.

## Introduction

Bisphosphonates are analogues of pyrophosphate preferentially taken up by skeletal sites of increased remodeling and slowly eliminated from bone; they suppress bone resorption by inhibiting posttranslational modification of important intracellular proteins essential for osteoclast function and survival. Denosumab is a fully human monoclonal antibody against receptor activator of nuclear factor-kβ (RANKL) which prevents RANKL (ligand)-RANK (receptor) interaction on surface of osteoclast precursors and mature osteoclasts, inhibiting osteoclast differentiation, function, and survival. Bisphosphonates and denosumab are commonly used and effective antiresorptive agents for managing postmenopausal osteoporosis, and they have demonstrated overwhelmingly favorable benefit/risk profiles regarding risk of osteoporotic fractures vs skeletal adverse events. External ear canal osteonecrosis is a rare skeletal complication associated with antiresorptive use. We describe 2 patients who developed external auditory canal osteonecrosis while taking antiresorptive therapy for osteoporosis.

## Case Presentation

**Figure 1.**
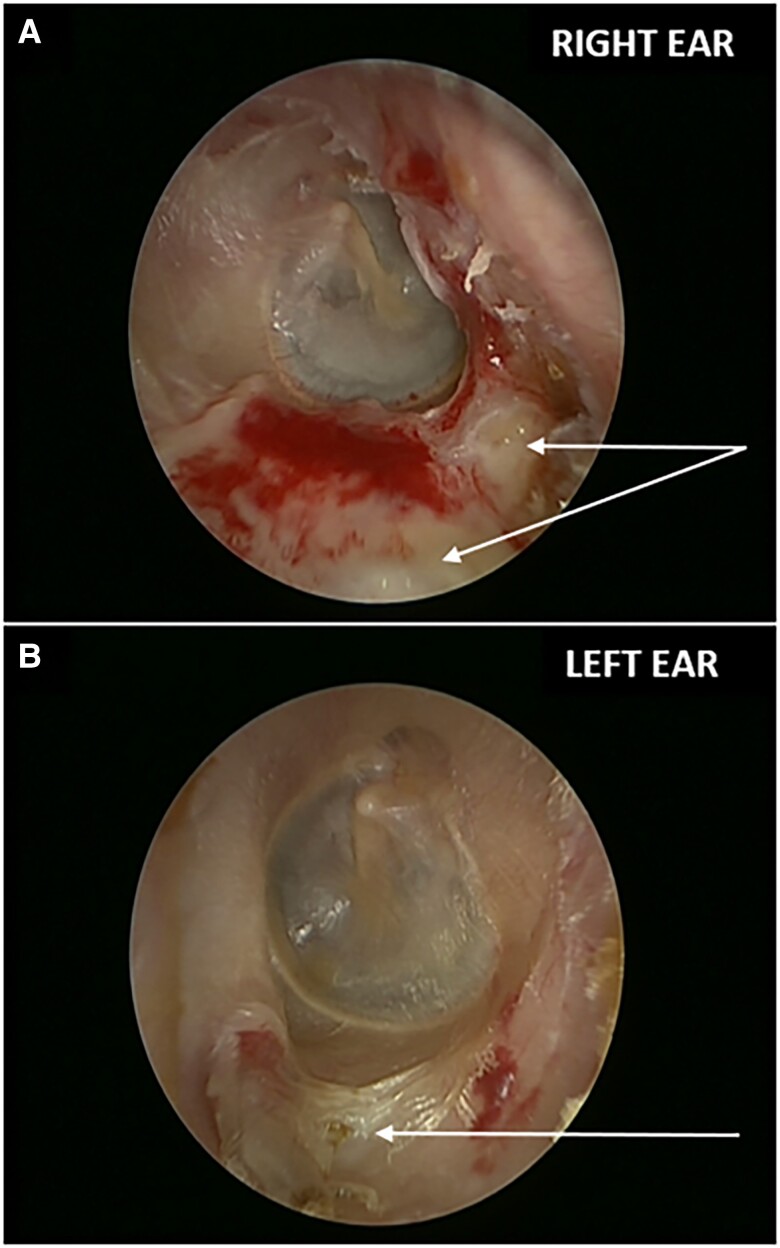
Otoscopic images indicating bilateral external auditory canal osteonecrosis. Otoscopy demonstrates nonhealing areas of exposed bone (white arrows) in the walls of right (A) and left (B) external auditory canals in a bisphosphonate-naïve 79-year-old woman with postmenopausal osteoporosis taking denosumab for 2 years.

Patient A: A 79-year-old postmenopausal woman commenced subcutaneous denosumab 60 mg every 6 months in 2018 for low lumbar spine 0.82 g/cm^2^ (T-score −3.1) and femoral neck bone mineral density of 0.76 g/cm^2^ (T-score −2.0). In 2020, she underwent clearance of right ear wax by an ear/nose/throat (ENT) specialist which incidentally revealed areas of exposed bone in both external auditory canals (Fig. 1). She had no preceding otorrhea, otalgia, or hearing loss and her facial nerve function was intact. She had no previous exposure to bisphosphonates, glucocorticoids, or local radiotherapy and no history of malignancy or diabetes mellitus. She is a nonsmoker, does not drink excessive alcohol and is not a habitual cotton bud user.

Patient B: A 64-year-old postmenopausal woman was diagnosed with osteoporosis in 2010 with several midthoracic vertebral compression fractures and artefactually “normal” lumbar spine 1.15 g/cm^2^ (T-score −0.4) and low femoral neck bone mineral density of 0.81 g/cm^2^ (T-score −1.4). She had received risedronate for 5 years followed by denosumab for 5 years. In 2020, she developed right-sided otalgia and an ENT specialist found the patient to have an area of exposed bone in the right external auditory canal (no figure available). She had no previous glucocorticoid use or local radiotherapy and no history of malignancy or diabetes. She is a nonsmoker, has no excessive alcohol intake and is not a habitual cotton bud user.

## Diagnostic Assessment

Patient A: Computed tomography (CT) of temporal bones did not find evidence of cholesteatoma, mass, or bone erosion. Bone turnover was suppressed consistent with antiresorptive therapy with low serum C telopeptide of type 1 collagen (CTX) 95 ng/L (95 pg/mL) (100-1000) and low procollagen type 1 amino-terminal propeptide-1 (P1NP) 11 µg/L (11 ng/mL) (15-115).

Patient B: CT of her temporal bones was unremarkable with no bone erosion. Bone turnover was suppressed consistent with long-term antiresorptive therapy with low-normal urine deoxypyridinoline/creatinine ratio 4.3 nmol/mmol (3.0-7.4) and low P1NP of 11 ug/L (11 ng/mL).

## Treatment

Patient A: She was managed conservatively with topical ointment and denosumab was ceased.

Patient B: She was managed with topical ointment and antibiotics and denosumab was ceased.

## Outcome and Follow-up

External auditory canal findings of exposed bone in both patients were stable at 12 months, with no evidence of re-epithelialization.

## Discussion

Osteonecrosis of jaw (ONJ) is an uncommon skeletal complication of long-term bisphosphonates in osteoporosis and even rarer with denosumab. Only 13 cases of ONJ occurred in the FREEDOM extension (5.2 per 10 000 participant-years), which included 4550 postmenopausal osteoporotic women with up to 10 years denosumab exposure. The underlying pathogenesis is not well-defined, but ONJ has been associated with periodontal surgery/infection and poor dental hygiene in individuals with antiresorptive-induced suppression of bone resorption and angiogenesis (and thus impaired remodeling and wound healing) [1]. Risk factors for ONJ include intravenous bisphosphonate therapy, oncological indication, duration of antiresorptive exposure, diabetes, tobacco, and excess alcohol exposure and glucocorticoid use [1]. ONJ is a clinical diagnosis defined as exposed bone in the maxillofacial region for > 8 weeks in the absence of local radiotherapy whereby radiographic and histopathological findings are supportive but not required for diagnosis [1], a definition endorsed by various institutions such as The American Association of Oral and Maxillofacial Surgeons, American Society for Bone and Mineral Research, American Society of Clinical Oncology, and International Task Force on Osteonecrosis of the Jaw.

Osteonecrosis of the external auditory canal associated with antiresorptive use has been scarcely published in the literature to-date. We performed a literature review using PubMed/Medline search terms “*ear canal*” AND “*osteonecrosis*”; “*auditory canal*” AND *osteonecrosis*”; “*ear*” AND “*osteonecrosis*”; “*bisphosphonate*” AND “*ear canal*” AND “*osteonecrosis*”; “*bisphosphonate*” AND “*auditory canal*” AND “*osteonecrosis*”; “*bisphosphonate*” AND “*ear*” AND “*osteonecrosis*; “*denosumab*” AND “*ear canal*” AND “*osteonecrosis*”; “*denosumab*” AND “*auditory canal*” AND “*osteonecrosis*”; and “*denosumab*” AND “*ear*” AND “*osteonecrosis*.” Articles were also sourced from reference lists and related article lists to acquire all previously published cases of antiresorptive-associated external auditory canal osteonecrosis. According to previously published cases and likely similar pathogenesis to ONJ, osteonecrosis of auditory canal has a working definition of exposed bone in the ear canal for > 8 weeks without previous exposure to local radiotherapy and is a clinical diagnosis whereby radiographic and histopathological findings are supportive but not required for diagnosis [2–10]. Including our cases, there have been 27 cases overall comprising 20 patients taking antiresorptives for osteoporosis (Table 1) [2–10] and 7 patients with an oncological indication such as myeloma or breast cancer. Regarding those taking antiresorptives for osteoporosis, all patients except 1 were female and from 64 to 89 years of age, consistent with the postmenopausal osteoporotic population commonly prescribed these agents. Bisphosphonates have been implicated in 18/20 cases of which 2 were bisphosphonate-naïve (including our case). The other case of bisphosphonate-naïve osteonecrosis of auditory canal was an 81-year-old woman with left-sided otorrhea after 18 months of denosumab exposure [4]. The duration of antiresorptive exposure prior to onset of symptoms or diagnosis has ranged between 1.5 years and > 15 years. Risk factors were not consistently reported, with 1 patient being an ex-smoker, 1 an active smoker, and 2 patients with preceding local trauma (ear toilet and habitual cotton bud use). No patients had reported history of local radiotherapy, systemic glucocorticoid use, diabetes, or excessive alcohol intake. Osteonecrosis has been bilateral in 50% of cases (7/14), indicating importance of assessing the other ear canal when unilateral findings are discovered. In cases where symptomatology was well-defined, the most common were otalgia (10/12), otorrhea (5/12), and deafness (3/12), with our case being the only patient described with asymptomatic auditory canal osteonecrosis incidentally discovered on otoscopy. In all cases, otoscopy demonstrated areas of exposed bone in the external auditory canal, sometimes with associated inflammatory changes. Bony destruction, inflammation, and/or erosion has been demonstrated on CT of temporal bones in 6/14 patients, with other patients either having unremarkable CT or CT was not required to make the diagnosis. Of the 5 patients who underwent biopsy of the lesions, all had supportive evidence on histopathology showing necrotic and inflammatory bony changes. Treatment in almost all cases involved conservative topical therapy initially (antibiotics and/or steroids) and cessation of antiresorptives. Outcomes have been varied and poorly defined, with some cases responding well to conservative therapy and others needing more extensive treatment, such as intravenous antibiotics, debridement or surgical resection of necrotic bone, and subsequent reconstruction. It is unclear, based on available literature, whether cessation of antiresorptives has any beneficial role in the outcome of this condition given this was performed in vast majority of cases, as well as whether recommencement of antiresorptives is safe once healing is complete. Given the rarity of antiresorptive-associated osteonecrosis of the external auditory canal, we do not recommend routine screening for pre-existing auditory canal disorders at commencement of antiresorptives but rather clinicians should be aware of this complication in case patients report new-onset ear-related symptoms while on treatment, such as otalgia, otorrhea, or hearing impairment.

**Table 1.** Summary of published cases of external auditory canal osteonecrosis in patients taking antiresorptives for osteoporosis (*n* = 14) [2–10]

Author (et al)	Age Sex	Antiresorptive duration	Risk factors	Location	Symptoms	Supportive tests	Treatment	Outcome
Kumar	79 F	Denosumab 2 years	Nil radiation, steroids, diabetes, trauma, smoking, or alcohol	Bilateral	Nil	Otoscopy	Topical BP ceased	Stable at 12 months
	64 F	Risedronate 5 years Denosumab 5 years	Nil radiation, steroids, diabetes, trauma, smoking, or alcohol	Unilateral	Otalgia	Otoscopy	Topical BP ceased	Stable at 12 months
Abelardo	71 F	Alendronate >5 years	Not reported	Bilateral	Otalgia Otorrhea Deafness	Otoscopy	Topical BP ceased	Healed at 6 months
	76 F	Alendronate >4 years	Not reported	Unilateral	Otalgia	Otoscopy	Topical BP ceased	Healed at 6 months
	83 F	Alendronate 6 years	Not reported	Bilateral	Otalgia Otorrhea	Otoscopy	Topical BP ceased	Healed at 6 months
True	79 F	Alendronate >10 years Denosumab 4 months	Ex-smoker Nil radiation, diabetes, or trauma	Bilateral	Otalgia Deafness	Otoscopy CT Biopsy	Topical	Completely healed at 1 year
Takeda	81 F	Denosumab 1.5 years	Nil radiation, steroids, or diabetes	Bilateral	Otorrhea Deafness	Otoscopy CT Biopsy	Topical BP ceased Surgery	No recurrence 1.5 years later
McCadden	69 to 89 5 F 1 M	BPs 20 months to >15 years	Nil radiation, steroids, or diabetes	Bilateral in 2 cases Not reported in other cases	Not reported	Otoscopy	Topical BP ceased in 5/6 cases Debrided in 1/6 cases	Completely healed at 1 year in 5/6 cases
Takahashi	71 F	Alendronate 11 years	Not reported	Unilateral	Otalgia	Otoscopy CT MRI Biopsy	BP ceased Debrided	Rapidly improved
Thorsteinsson	76 F	Alendronate 8 years Ibandronate 3 years Zoledronic acid 3 years	Active smoker (25 pack years) Nil radiation, steroids, diabetes, or trauma	Bilateral	Otalgia	Otoscopy CT	Topical BP ceased Debrided Steroids IV Abx Surgery	N/A
Wickham	72 F	Alendronate 3.5 years	Ear toilet Nil diabetes, steroids, or trauma	Unilateral	Otalgia	Otoscopy CT Biopsy	Topical BP ceased Surgery	N/A
Kharazmi	74 F	Alendronate N/A	Not reported	Bilateral	Otalgia Otorrhea	Otoscopy	Topical BP ceased	Stable at 7 months
	67 F	Alendronate 2 years	Not reported	Unilateral	N/A	Otoscopy	Topical	Stable
	88 F	Alendronate Several years	Not reported	Unilateral	N/A	Otoscopy	N/A	N/A
Salzman	79 F	Alendronate 10 years	Habitual cotton bud use Nil radiation, steroids, diabetes, trauma, smoking, or alcohol	Unilateral	Otalgia Otorrhea	Otoscopy CT Biopsy	Topical BP ceased IV Abx Debrided	Started to heal at 3 months

Abbreviations: Abx, antibiotics; BP, bisphosphonates; CT, computed tomography; IV, intravenous.

External auditory canal osteonecrosis is a rare skeletal complication that can occur in patients taking bisphosphonates for osteoporosis as well as denosumab without prior bisphosphonate exposure. The rarity of this disorder in reported literature may be compounded by the lack of awareness in clinical practice among physicians prescribing antiresorptive agents. Development of otorrhea, otalgia, and/or hearing loss while on antiresorptive therapy should alert physicians to this rare clinical entity and prompt ENT surgical referral to facilitate early diagnosis and management.

## Learning Points

- We present 2 patients who developed external auditory canal osteonecrosis while taking denosumab for postmenopausal osteoporosis, one of whom was bisphosphonate-naïve and the other symptomatic with otalgia. There have been 12 other cases reported in patients with osteoporosis.
- In both cases, otoscopy by an ENT surgeon revealed areas of exposed bone in the external auditory canal.
- Localizing ear symptoms in patients taking antiresorptives should alert physicians to this rare entity and prompt ENT surgical referral for prompt diagnosis and initiation of management.

## Contributors

S.K. and T.D. conceptualized the manuscript. S.K. collected the data, interpreted the data, and drafted the manuscript. T.D. and J.W. revised the manuscript critically. S.K., T.D., and J.W. approved of the final version of the manuscript and agree to be accountable for all aspects of the work. S.K. is the guarantor of the manuscript.

## Data Availability

Data sharing is not applicable to this article as no datasets were generated or analyzed during the current study.

## Abbreviations

CT: computed tomography
ENT: ear/nose/throat
ONJ: osteonecrosis of the jaw
P1NP: procollagen type 1 amino-terminal propeptide-1
